# Study Hypoxic Response under Cyclic Oxygen Gradients Generated in Microfluidic Devices Using Real-Time Fluorescence Imaging

**DOI:** 10.3390/bios12111031

**Published:** 2022-11-17

**Authors:** Dao-Ming Chang, Yi-Chung Tung

**Affiliations:** 1 Research Center for Applied Sciences, Academia Sinica, Taipei 115, Taiwan; 2 College of Engineering, Chang Gung University, Taoyuan 333, Taiwan

**Keywords:** hypoxic response, cyclic oxygen gradient, microfluidic device, fluorescence imaging

## Abstract

Oxygen plays important roles in regulating various biological activities under physiological and pathological conditions. However, the response of cells facing temporal variation in oxygen microenvironments has seldom been studied due to technical limitations. In this paper, an integrated approach to studying hypoxic response under cyclic oxygen gradients is developed. In the experiments, a cell culture system based on a microfluidic device is constructed to generate cyclic oxygen gradients with desired periods by alternately introducing gases with specific compositions into the microfluidic channels next to the cell culture channel separated by thin channel walls. Observation of the hypoxic responses is performed using real-time fluorescence imaging of dyes sensitive to extra- and intracellular oxygen tensions as well as intracellular calcium concentrations. Cellular hypoxic responses of human aortic smooth muscle cells (AoSMCs) and lung carcinoma epithelium (A549) cells, including intracellular oxygen and calcium levels, are measured. The results show that the two types of cells have different hypoxic responses to the applied cyclic oxygen gradients. With the capability of real-time cellular response monitoring under cyclic oxygen gradients, the developed approach provides a useful scheme to investigate hypoxic responses in vitro under microenvironments mimicking various in vivo physiological and pathological conditions.

## 1. Introduction

Molecular oxygen (O_2_) is essential for cell survival. While the atmospheric oxygen concentration remains constant at around 20.9%, the concentration among different parts of human tissues can be as high as 19.7% in the tracheas or as low as about 1% in the superficial region of the skin [1]. When oxygen availability is compromised, cells act accordingly to sustain life. The collective actions are called hypoxic responses [2,3,4,5,6,7,8,9]. Studying the hypoxic response and the underlying oxygen-sensing mechanism is therefore important. In both physiological and pathological conditions, hypoxic responses play key roles in regulating various biological activities. For instance, hypoxic pulmonary vasoconstriction (HPV) is one of the essential physiological hypoxic responses. Pulmonary arteries constrict in response to alveolar hypoxia to improve gas exchange in the lungs and blood delivery to systemic tissues [10]. 

In addition, tumor-induced hypoxia is observed among various types of cancers, with an average 1.5% to 2% of oxygen tensions in cancer biology. Tumor-induced hypoxia has been proven to enhance progression, metastasis, angiogenesis and therapeutic resistance [11]. As a result, targeting the hypoxic signaling mechanisms of cancer cells is believed to possess therapeutic values. In chronic hypoxia in the core region of a solid tumor, drug targeting of the hypoxia-induced factor (HIF-1α) has been proven to be a promising therapeutic strategy [12,13]. Moreover, under cyclic hypoxia conditions in which the cell experiences intermittent hypoxia and normoxia environments, recent studies show HIF-2α is the dominant signaling factor for the fact that prolonged (>4–12 hr) cyclic hypoxic/normoxic cycles enhance HIF-2α but not HIF-1α accumulation among different cancer cell lines [14]. This observation suggests that different signal transduction mechanisms may exist for chronic and intermittent hypoxia.

In order to study the hypoxic response and oxygen-sensing mechanism of cells, precise and consistent control of extracellular oxygen tension in spatiotemporal domains is highly desired. In conventional broadly conducted cell culture experiments, cells are cultured using a bicarbonate-buffered medium at 37 °C with 5% CO_2_ enriched ambient air and humidity under non-condensing conditions in incubators. When performing hypoxic experiments using a gas control incubator/chamber, the typical response time taken to reach a steady state is within several tens of minutes to an hour, depending on the chamber size and setpoint value. After the culture environment reaches the setpoint, it further takes several additional minutes to hours to equilibrate the dissolved oxygen into the gas phase [15]. Despite the gas-controlling incubation/chamber proving to be useful for chronic hypoxic research over the last 2 decades, a cell culture system capable of manipulating oxygen tension at faster responses is needed to explore acute hypoxic response and sensing mechanisms of the cells.

To overcome the challenges, an approach capable of reliably altering extracellular environments between different hypoxia levels and the normoxia condition with a relatively short response time is needed. Among various methods, microfluidics has been broadly exploited for cell culture in dynamic microenvironments due to its desired advantages, including a small sample volume and a high surface area to volume ratio which can greatly help to reduce the response time required to reach an equilibrium. For example, Polinkovsky et al. developed a microfluidic device capable of generating a series of oxygen concentrations with a short response time (in the order of seconds) through on-chip gas mixing to study cellular responses [16]. In addition, Chen et al. exploited the spatially-confined chemical reaction method to rapidly establish oxygen gradients within the microfluidic channel within minutes for cell culture applications [17]. The same chemical reaction method has also been applied to investigate cancer cell migration, endothelial cell migration and three-dimensional (3D) network formation under various oxygen microenvironments [18,19].

However, the existing approaches often require complicated instrumentation and do not provide great real-time cellular analysis capabilities, which are critical to studying temporal cellular responses. As a result, an integrated approach combining a microfluidic device, fluorescence dyes sensitive to extra/intracellular oxygen tensions and calcium concentrations, and real-time fluorescence imaging is developed in this paper to investigate temporal cellular hypoxic responses under various cyclic oxygen tensions. In the experiments, a single-layered microfluidic device capable of generating cyclic oxygen gradients and switching between hypoxic oxygen gradients and normoxic conditions within minutes is constructed for cell culture. The device possesses a simple architecture while allowing easy access for optical imaging and sensing. The device is mainly made of an elastomeric material, polydimethylsiloxane (PDMS) (due to its desired gas permeability), optical transparency, and cell compatibility. In the developed device, the oxygen gradient is generated by flowing gas with specific compositions into a microfluidic channel next to the cell culture channel separated by thin walls. The gas flow is controlled using a computer-controlled three-way solenoid valve. The single-layer device architecture with a good optical transparency of PDMS and a glass substrate makes real-time observations of the acute hypoxic response of cells using microscopes feasible.

For the hypoxic response study, two types of cells, smooth muscle cells and epithelial cells, were cultured inside the devices in the cell experiments for demonstration. The function of the device was first characterized to confirm its capability for cyclic oxygen gradient generation. The intracellular oxygen tensions and calcium levels were then measured in a time-lapse manner for the two distinct cell types under the generated cyclic oxygen gradients. The experiment results confirmed the capability of the device to generate the cyclic oxygen gradients for the cell experiments. Furthermore, the cell experiments showed the application of real-time fluorescence imaging for the study of cellular hypoxic responses under the cyclic oxygen gradients in variation of the intracellular oxygen tensions and calcium levels. The cell experimental results indicated distinct responses of the two tested cell types and suggested the importance of investigating hypoxic responses of the cells in the temporal domain. With the short response time, high throughput on control of oxygen tensions and high compatibility to optical assays of the device, the developed approach integrating the real-time fluorescence imaging serves as an efficient method to explore cell kinetic responses under extracellular oxygen tension variation as well as critical oxygen tension triggering acute hypoxic responses and underlying mechanisms for various biomedical studies.

## 2. Materials and Methods

### 2.1. Device Design and Fabrication

**Figure 1:**
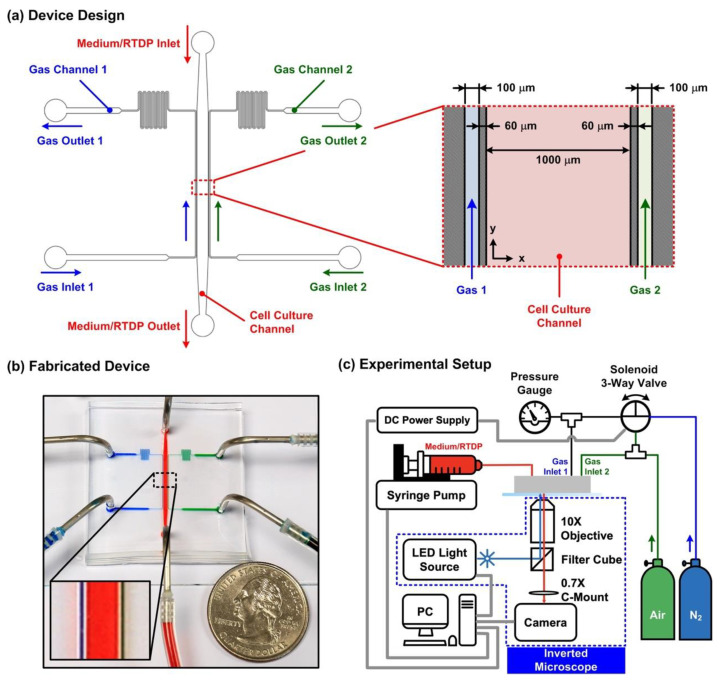
(**a**) Schematic of the PDMS microfluidic device capable of generating cyclic oxygen gradients. Three sets of microfluidic channels were designed in the device, including the middle cell culture channel and two side channels for oxygen gradient generation. The channels are separated by 60 μm-wide walls. Oxygen gradient in the cell culture channel is generated by purging pure nitrogen gas and air into the left (Inlet 1) and right (Inlet 2) side channels, respectively. (**b**) Experiment photos of the fabricated device filled with food dyes. (**c**) Experiment setup for generation of the cyclic oxygen gradients using the microfluidic device for cell culture experiments and observation.

The microfluidic device capable of generating cyclic oxygen gradients is made of PDMS, and it is composed of a top layer with microfluidic channel patterns and a PDMS-coated glass bottom layer. Three sets of microfluidic channels were designed in the top layer, including one 1 mm-wide middle channel for cell culture and two sets of 100 μm-wide side channels (Figure 1a). The channels were all designed with single inlets and outlets. Air or nitrogen gas was injected into the side channel from the inlet for the cyclic oxygen gradient generation. The middle and side channels are separated by 60 μm-wide oxygen-permeable PDMS thin walls to prevent direct chemical contact and gas bubbling into the medium while altering oxygen tensions in the middle channel.

The top layer was fabricated using the well-developed soft lithography replica molding process. A master mold with positive relief features of the microfluidic channels was fabricated on a 4-inch silicon wafer using negative tone photoresist (SU-8 2050, MicroChem Co., Newton, MA, USA) via conventional optical photolithography. To prevent the adhesion of the cured PDMS onto the mold, the fabricated mold was silanized using 1H,1H,2H,2H-perfluorooctyltrichlorosilane (78560-45-9, Alfa Aesar, Tewksbury, MA, USA) for 30 min in a desiccator at room temperature. A PDMS pre-polymer with 1:10 (*w*/*w*) of curing agent and base was mixed, poured onto the mold, degassed in a desiccator, and then cured at 60 °C for more than 4 h. After curing, the interconnection inlets and outlets were created via a biopsy punch with a diameter of 1.5 mm. For the bottom layer fabrication, a glass slide was spin-coated with the aforementioned PDMS pre-polymer and cured at 60 °C for more than 4 h. Both layers were treated with oxygen plasma (PX-250, Nordson MARCH Co., Concord, CA, USA) at 95 W for 35 s, sealed against each other, and heated at 60 °C for more than 4 h to promote the bonding between the layers. Figure 1b shows the experiment photos of the fabricated devices filled with food dyes. The simple device design and fabrication process make the device easy to set up for practical biological applications.

### 2.2. Oxygen Gradient Generation and Characterization

In order to generate cyclic oxygen gradients within the middle cell culture channel, mixed air (air with 5% CO_2_) was constantly introduced into one side channel and pure nitrogen (i.e., 0% O_2_) and the mixed air were alternately introduced into another side channel with a period of 10 min. Both mixed air and nitrogen gases were injected into the side channels with inlet pressures of 0.4 bar controlled via manual gas regulators. The alternation between the gases was accomplished by switching the power of a three-way solenoid valve (ZHV-0519, ZonHen Co., Ltd., Shenzhen, China) on/off using a personal computer-controlled DC power supply (E3631A, Agilent Technologies, Inc., Santa Clara, CA, USA) via a GPIB interface, as shown in Figure 1c. In the experiments, the solenoid valve was operated under 5 V/0.2A. The photo of the experimental setup is shown in Figure S1 in the Supplementary Information. By introducing the gas with a specific oxygen composition into the side channel, the oxygen tension in the ambient air surrounding the channel can be adjusted to that of the introduced gas. The method has been broadly applied to control cell culture oxygen microenvironments in various microfluidic devices with relatively simple instrumentation [20].

In order to characterize the oxygen tensions generated in the middle cell culture channel, an oxygen-sensitive fluorescence dye, Tris(2,2′-bipyridyl)dichlororuthenium(II) hexahydrate (RTDP) (224758, Sigma-Aldrich, Inc., St. Louis, MO, USA) was perfused into the middle channel with a flow rate of 10 μl/min. For the RTDP intensity profile measurement, 25% power blue LED illumination, 200 ms exposure, no average and binning were set as imaging parameters. The fluorescence intensity of the dye was quenched by the presence of oxygen; therefore, the spatial distribution of the oxygen tension inside the channel can be estimated by measuring the intensity profile [21]. The relationship between the fluorescence intensity and oxygen tension is given by the Stern-Volmer equation as $I_{f}^{0}/I_{f}=1+K\cdot \left[Q\right]$, where $I_{f}$ and $I_{f}^{0}$ are the fluorescence intensity values with and without quencher (oxygen), respectively. K represents the quenching coefficient, and [Q] is the concentration of the quencher (oxygen). The quenching coefficient K was calibrated by introducing pure nitrogen and oxygen, representing 0% and 100% oxygen tension, into the side channels, respectively [17]. The calibrated quenching coefficient was further validated by calculating the oxygen tension under mixed air gas (ambient air with 5% CO_2_). Since oxygen tension in air is approximately 20.9%, the oxygen tension in the mixed air should be around 20.9 × 95% = 19.9% with an additional 5% CO_2_ mixed in the air. The resulted oxygen tension needs to be close to 20% to ensure the proper estimation of the quenching constant for the accurate oxygen tension characterization in the device.

### 2.3. Cell Culture and Seeding

To demonstrate the capability of the integrated approach, two types of cells, smooth muscle cells and epithelial cells, were cultured and investigated in the experiments. First, a human aortic smooth muscle cell (AoSMC) (CC-2571, Lonza, Basel, Switzerland) was exploited. The cells were cultured in a smooth muscle cell basal medium (CC-3182, Lonza) with growth factor supplements (CC-4149, Lonza) and maintained as per the following process. The attached cells were trypsinized with trypLE (12604013, Gibco), centrifuged at 209× *g* for 5 min and resuspended in 1 mL of the prewarmed medium. After cell counting, 7 × 10^4^ cells were seeded into a 25 cm^2^ flask (156367, Thermo Fisher Scientific, Waltham, MA, USA). The medium was changed every 2–3 days, and the cells were passaged when they reached 70–80% confluency, which took around 1 week. For optimized cell conditions and consistent results, the AoSMCs used in the experiments were all under 10 passages. In addition, human alveolar basal epithelial cells (A549, ATCC, Manassas, VA, USA) were also cultured in the experiments for comparison. The cells were cultured in DMEM (Gibco 10566, Thermo Fisher Scientific) with 10% *v*/*v* FBS (Gibco 10082, Thermo Fisher Scientific) and 1% *v*/*v* antibiotics (Gibco 15240, Thermo Fisher Scientific). The cell stocks were all maintained in a 37 °C humidified incubator supplied with 5% CO_2_. Cell suspension used for the cell culture in the devices was prepared by a process similar to the routine passage, except that after centrifugation, cell suspension was concentrated by resuspending the pellets with 100 μL of the prewarmed medium. After cell number counting, concentrated cell suspension was further diluted into final concentrations of 5000 cells/μL and 10,000 cells/μL for AoSMC and A549, respectively.

Before seeding the cells into the microfluidic device, the cell culture channel was first made hydrophilic through oxygen plasma treatment at 95 W for 35 s. The channel was then coated with a cellular matrix protein, fibronectin (FC010, Merck Millipore, Burlington, MA, USA), at a concentration of 100 μg/mL to promote cell attachment. The cell suspension was then pipetted into the cell culture channel from the inlet. Approximately 20 μL of cell suspension was needed for single device seeding. The cells were cultured inside the device for more than 18 h under static conditions to ensure the proper adhesion of the cells to the substrate for the following experiments.

### 2.4. Fluorescence Imaging Analysis

For the study of cellular responses under cyclic oxygen gradients, two types of analyses were performed in the experiments. First, the intracellular oxygen tension was characterized using a commercially available MitoXpress Intra intracellular oxygen assay (MX-300, Agilent Technologies, Inc.). The reagent was diluted with the complete cell culture medium at a volumetric ratio of 1:10 and applied into the cell-seeded device while the cells were attached to the culture surface. The cells were further incubated in the diluted reagent overnight. 

In addition, intracellular calcium concentration was also investigated in the experiments. Calcium ion (Ca^2+^) is an essential intracellular messenger regulating various cell functions. Monitoring calcium levels under temporal and spatial oxygen tension change potentially gives insights into understanding the fast-acting oxygen sensing mechanism and hypoxic response behind it. In the Ca^2+^ measurement experiments, after the cell seeding and overnight culture, the growth medium inside the device was replaced by the complete medium with 3 μM of X-Rhod-1 Acetoxymethyl (AM) ester (Invitrogen X14210, Thermo Fisher Scientific). The cells were incubated for 30 min at 37 °C for dye loading. After the dye loading, the cells were then washed using the complete medium and incubated at 37 °C for an additional 30 min to make AM ester completely hydrolyzed and thus make X-Rhod-1 fluoresces.

The stained cells cultured in the microfluidic devices were imaged using an inverted fluorescence microscope (DM IL, Leica Microsystems, Wetzlar, Germany) equipped with a CCD camera (DFX360, Leica Microsystems, Wetzlar, Germany), a multi-color LED light source (X-Cite XLED1, Excelitas Technologies Corp., Waltham, MA, USA), and a 10X objective (HI Plan, Leica Microsystems). Control of the microscope and automatic time-lapse image acquisition were achieved through a commercially available microscopy automation and image analysis software (Metamorph Version 7.7, Molecular Devices, LLC, San Jose, CA, USA). For the intracellular oxygen characterization, a multiband filter cube suitable for MitoXpress Intra imaging (380–395 nm band-pass excitation filter, 575 nm dichromatic mirror, and 580–650 nm band-pass emission filter) was exploited (Semrock DA-FI-TR-3X-A-000, IDEX Health & Science, Rochester, NY, USA) (Figure 1c). The imaging parameters were set as UV LED excitation at 5% intensity, 200 ms exposure, 4 times frame averaging and 2 × 2 binning. To minimize the photocytotoxicity during the imaging, the images were captured every 1 min for an hour. For the intracellular calcium observation, the same multiband filter cube with a blue band-pass excitation filter (470–490 nm), 500 nm dichromatic mirror and 500–535 nm band-pass emission filter was exploited. The imaging parameters were set as blue LED excitation at 5% intensity, 200 ms exposure and 2 × 2 binning. For AoSMC and A549, the images were captured every 2 s and 30 s for an hour, respectively.

The captured time-lapsed fluorescence images were quantitatively analyzed using the imaging analysis software ImageJ (Ver. 1.52p, NIH) with Time Series Analyzer plugin (https://imagej.nih.gov/ij/plugins/time-series.html Access Date: 15 August 2020) and further visualized via a mathematics software, MATLAB (R2017a, MathWorks, Natick, MA, USA). All time-lapsed fluorescence intensity values were reported through fold change compared to the intensity obtained from the first frame in the same experiment (normalized fluorescence intensity, F_i_/F_0_). For intracellular oxygen measurements, baseline correction was applied by fitting the time-lapsed normalized fluorescence intensity data against asymmetric truncated quadratic function to compensate for the effect of photobleaching.

## 3. Results and Discussion

### 3.1. Characterization of Cyclic Oxygen Gradient Generation

The oxygen gradient was generated and eliminated by alternately introducing the mixed air (air with 5% CO_2_) (N_2_ OFF) and pure nitrogen (N_2_ ON) into the left side channel while constantly flowing the mixed air into the right side channel in the experiments. The control of alternation between the gases was achieved using the computer-controlled three-way solenoid valve (Figure 1). 

**Figure 2:**
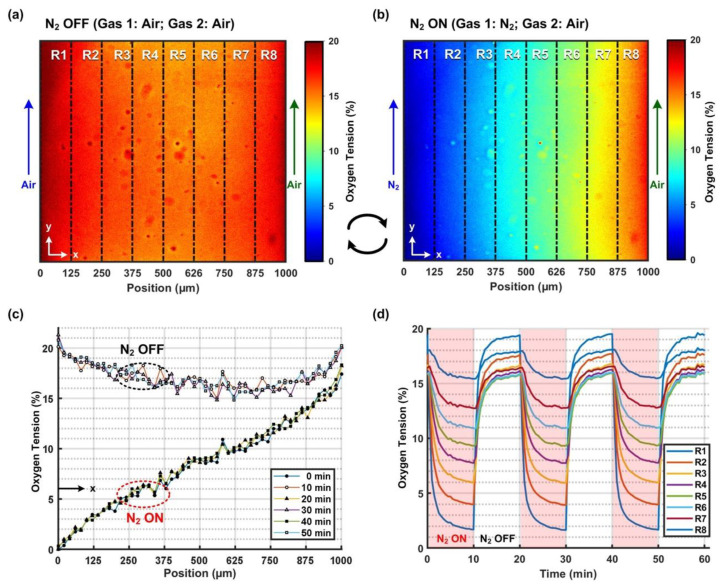
(**a**,**b**) are the characterized oxygen profiles without (N_2_ OFF) and with (N_2_ ON) oxygen gradients, respectively. (**c**) Line profiles show that consistent oxygen gradients (solid symbol lines) can be established when nitrogen gas is on (N_2_ ON), and relative uniform normoxia conditions (hollow symbol lines) can be recovered by air purging (N_2_ OFF). (**d**) The temporal variation in average oxygen tension values from the leftmost (R1) to the rightmost region (R8). Cyclic oxygen tension profiles in different regions show that the oxygen tensions oscillate in the period of 20 min (10 min on/10 min off).

The oxygen tension profiles characterized using the oxygen-sensitive dye and calculated based on the Stern-Volmer equation in the cell culture channel are shown in Figure 2. Figure 2a,b show the oxygen tension distribution within the cell culture channel when introducing the mixed air and the pure nitrogen into the left side channel, respectively. An animation of the real-time oxygen tension profile change in the cell culture channel is available in the Supplementary Information (Video S1). When flowing the mixed air into both side channels, the oxygen tension profile was more uniform across the width of the cell culture channel with an average value of 17.2%, as shown in Figure 2a. The majority of the oxygen tension within the channel (>90% of the area) lay between 17 and 20%, and the lowest value occurred at the center position of the channel due to a long diffusion length. In contrast, after switching from the mixed air to the nitrogen gas in the left side channel, the oxygen tension within the cell culture channel monotonically decreased from the right to left sides, ranging from 0.3% to 18.3%, as shown in Figure 2b. The plot shows that the oxygen gradient can be successfully established using the developed device and method. During the cyclic oxygen gradient generation process, the two oxygen tension profiles were switched every 10 min.

In order to quantify the generated cyclic oxygen gradients and their temporal patterns, the oxygen tension profiles along the channel width at different time points under the N_2_ OFF and ON conditions are plotted in Figure 2c. The plot shows that the oxygen tension profiles are consistent under both conditions, and the differences between the profiles under the N_2_ OFF and N_2_ ON conditions are less than 0.83% and 0.64%, respectively. To further observe the dynamics of the oxygen tension variation in the temporal domain, the cell culture channel was divided into eight regions for quantitative analysis. The temporal variation in the average oxygen tension within each zone is plotted in Figure 2d. The plot shows that the average oxygen tension cycled between 19.5% and 1.6% within the region closest to the left side channel (R1). In contrast, for the most distal region from the left side channel (R8), the oxygen tension cycled between 18.1% and 15.5%. The average oxygen tension values varied from different hypoxia degrees, ranging from 1.6% to 19.5% from the left (R1) to right (R8) side of the channel to normoxia (higher than 18.9%) in a period of 20 min. In addition, the temporal variation patterns in all regions within each period were similar to those in other periods. The results indicate that consistent cyclic oxygen gradients can be successfully generated using the device and further exploited for cell culture applications.

### 3.2. Intracellular Oxygen Tension Characterization

**Figure 3:**
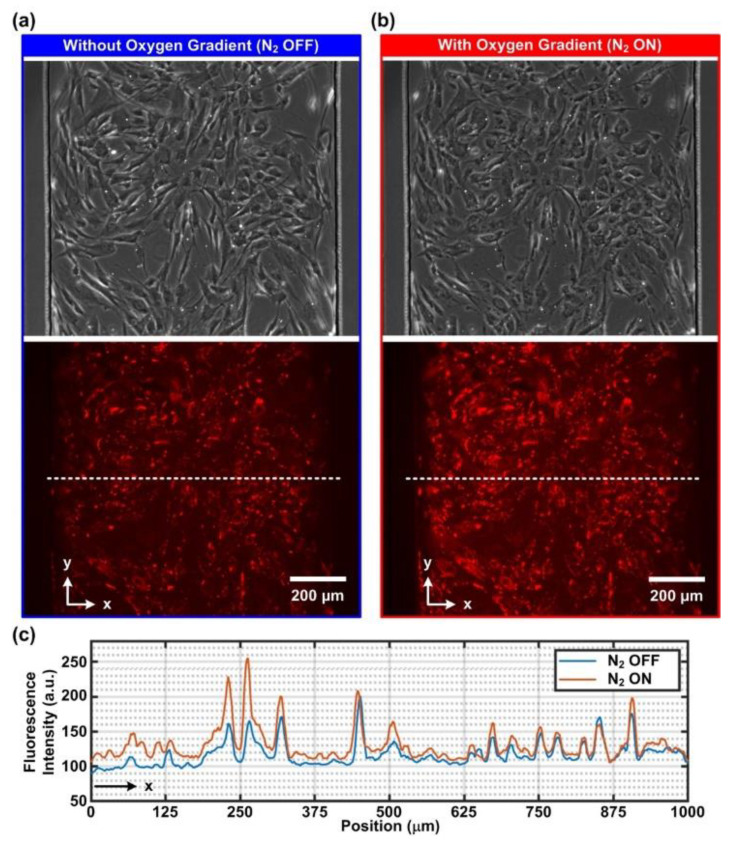
Phase contrast and fluorescence images of AoSMCs treated with the intracellular oxygen-sensitive reagent (MitoXpress Intra) under (**a**) normoxia (N_2_ OFF) and (**b**) oxygen gradient (N_2_ ON) conditions. (**c**) Representative line profiles of the fluorescence intensity across the width of the channel under the normoxia and oxygen gradient conditions. Compared to the normoxia condition (N_2_ OFF), higher fluorescence intensity on the left-hand side under the oxygen gradient condition (N_2_ ON) indicates lower intracellular oxygen tension within the AoSMCs.

**Figure 4:**
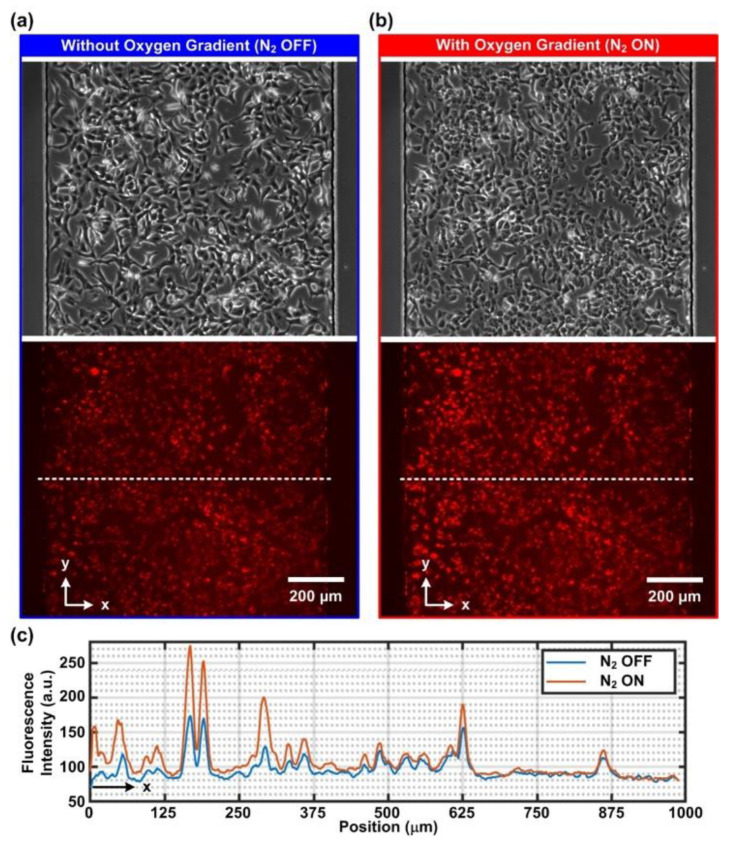
Phase contrast and fluorescence images of A549 cells treated with the intracellular oxygen-sensitive reagent (MitoXpress Intra) under (**a**) normoxia (N_2_ OFF) and (**b**) oxygen gradient (N_2_ ON) conditions. (**c**) Representative line profiles of the fluorescence intensity across the width of the channel under the normoxia and oxygen gradient conditions. Compared to the normoxia condition (N_2_ OFF), higher fluorescence intensity on the left-hand side under the oxygen gradient condition (N_2_ ON) indicates lower intracellular oxygen tension within the A549 cells.

For real-time observation of intracellular oxygen tensions, AoSMCs and A549 cells were stained with a fluorescent intracellular oxygen probe (MitoXPress Intra). The fluorescence of the probe was quenched by molecular oxygen; therefore, a decrease in fluorescence intensity indicated an increase in intracellular oxygen tension. Figure 3 and Figure 4 show the phase contrast and fluorescence images and quantitative fluorescence intensity profiles along the width of the channel in the AoSMC and A549 cell culture experiments, respectively. The phase contrast and fluorescent images were taken before (Figure 3a and Figure 4a) and after (Figure 3b and Figure 4b) the 1 h time-lapsed imaging process with 1 min intervals. Comparing the images before and after the 1 h imaging, it was observed that both cells remained attached to the substrates. However, a slightly rounded feature of the cells was observed after the measurements. The feature was suspected to be the phototoxicity effect caused by the probe/molecular oxygen quenching process which is known to produce reactive oxygen species (ROS, e.g., singlet oxygen) [22].

Furthermore, the fluorescent images under both N_2_ OFF (normoxia) and N_2_ ON (oxygen gradient) conditions were quantitatively analyzed, and the fluorescence intensity profiles are plotted in Figure 3c and Figure 4c for the AoSMCs and A549 cells, respectively. The plots show that the fluorescence intensity of the oxygen-sensitive dye was higher for the cells closer to the left side channel under the oxygen gradient condition when compared to the normoxic control ones. This suggests the lower intracellular oxygen tension of the cells on the left side when the N_2_ is on. In contrast, the fluorescence intensity for the cells close to the right side channel remained similar for both conditions, indicating a similar intracellular oxygen tension. The results confirmed that the intracellular oxygen tensions varied from low to high from left to right, following the trends of the oxygen gradient generated in the cell culture channel.

**Figure 5:**
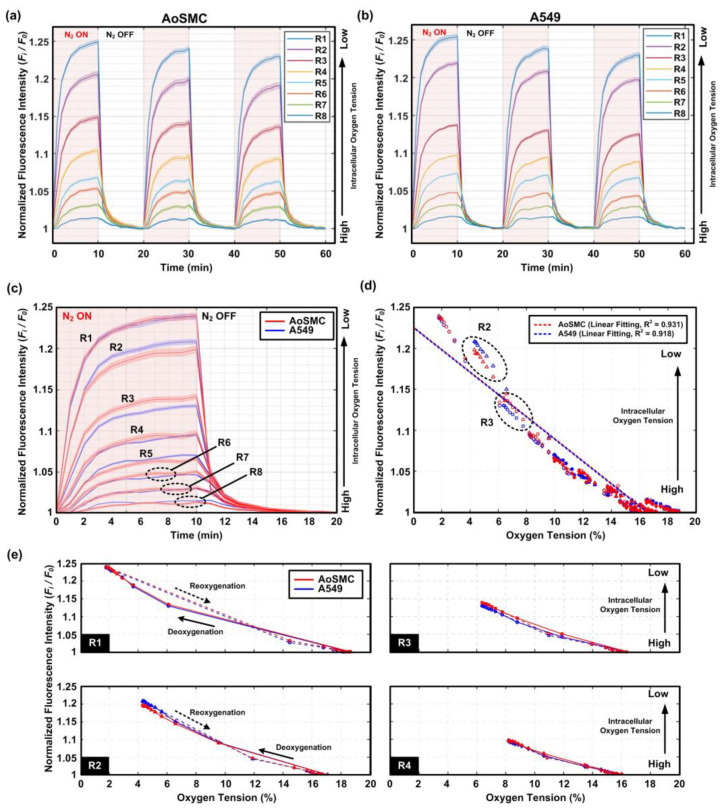
Temporal variation in the average normalized fluorescence intensities with the confidence bands (95% confidence interval) of the intracellular oxygen probes within the (**a**) AoSMCs and (**b**) A549 cells located in different regions across the width of the cell culture channel (left to right is R1 to R8, respectively). Red background regions indicate the periods when nitrogen gas is on (N_2_ ON) for the oxygen gradient generation. (**c**) Comparison of the average normalized fluorescence intensities with the confidence bands (95% confidence interval) of the intracellular oxygen probes within the AoSMCs and A549 cells located in different regions (R1 to R8) during the period of one oxygen gradient/normoxia (N_2_ ON/N_2_ OFF) cycle. Intracellular oxygen probe shows discrepant fluorescence intensity between the AoSMCs and A549 cells located in R2 and R3. (**d**) Plot of the normalized fluorescence intensities of the intracellular oxygen probes under different oxygen tensions generated in the microfluidic device for the cultured AoSMCs and A549 cells. (**e**) The kinetics of the intracellular oxygen tension of the cells located in regions R1 to R4 during the deoxygenation and reoxygenation processes within a single period based on the fluorescence intensity observation. The solid and dotted lines show the intracellular oxygen tension responses during deoxygenation (N_2_ ON) and reoxygenation (N_2_ OFF) processes, respectively.

In order to quantify the intracellular oxygen tension variation during the alternative normoxia (N_2_ OFF) and oxygen gradient (N_2_ ON) conditions, the average intensities of the oxygen-sensitive dye within the cells located in eight different regions across the channel width (left to right: R1 to R8) during the experimental period were plotted, as shown in Figure 5a,b for AoSMCs and A549 cells, respectively. The figures show that the normalized fluorescence intensity (F_i_/F_0_) of the intracellular oxygen tension probe increased when the gas introduced into the left side channel changed from the mixed air to N_2_, i.e., resulting in a change from the normoxia to oxygen gradient conditions in the cell culture channel. The intensity increase indicates that the intracellular oxygen tension decreased due to the surrounding extracellular environmental oxygen tension decrease. In addition, the fluorescence intensities within the cells located in the regions closer to the left side-channel with smaller region numbers were higher compared to those within the cells in the regions further away from the left side-channel under the oxygen gradient condition. The results indicate the cells have lower intracellular oxygen tensions when exposed to microenvironments with lower oxygen tensions, suggesting that the intracellular oxygen tensions of both AoSMCs and A549 cells can be modulated by the environmental oxygen microenvironments.

In order to compare the temporal hypoxic response of AoSMCs and A549 cells, the normalized fluorescence intensities within the cells located in different regions within a period of condition alternation are plotted in Figure 5c. The plots show that both cells located in regions 1 and 4–8 had similar intracellular oxygen tension responses with similar normalized fluorescence intensity variation patterns. Interestingly, the fluorescence intensities of the oxygen-sensitive probes within the cells located in regions 2 and 3 were slightly different between the two cell types. In region 2, the normalized intensity within the A549 cells rose slightly faster and reached a higher endpoint value than that within the AoSMCs when introducing the nitrogen into the side channel for oxygen gradient generation. In contrast, in region 3, the intensity within the A549 cells rose slightly slower and reached a lower endpoint value than that within the AoSMCs during the nitrogen introduction. The results suggest that the A549 cells reach higher and lower intracellular hypoxia levels in regions 2 and 3 compared to the AoSMCs, respectively. In addition, the results indicate a critical hypoxia level at which the two types of cells respond to the extracellular oxygen microenvironments differently.

For identification of the critical oxygen tension value, the normalized fluorescence intensity within the AoSMCs and A549 cells under different extracellular oxygen tensions during the generation of cyclic oxygen gradients was plotted, as shown in Figure 5d. In the figure, the *x*-axis plots the oxygen tension values calculated based on the oxygen-sensitive dye intensity measurement results performed in the oxygen gradient characterization experiments at specific time points, and the *y*-axis plots the normalized intracellular oxygen-sensitive dye fluorescence intensities at the same time points. The plot shows that most of the intensities within the two cell types overlapped for most of the oxygen tension values, and the relationships between the intensity and the oxygen tension are highly linear (R^2^ = 0.931 and 0.918 for linear fitting of data from the AoSMCs and A549 cells, respectively) for both cell types. The linear relation suggests that both cells vary their intracellular oxygen tension according to the extracellular oxygen microenvironments. Interestingly, the two cell types showed different fluorescence intensities when the extracellular oxygen tension was between approximately 4% and 8%, which was the oxygen tension within the aforementioned regions 2 and 3 during the oxygen gradient generation periods. In addition, the oxygen tension of approximately 6% was the value making the two cell types respond differently to the extracellular oxygen microenvironments. In future, a detailed study is required to better investigate the underlying molecular mechanisms resulting in the different intracellular oxygen responses between the two cell types. The study may further help biologists understand the temporal hypoxic responses of the cells to the extracellular oxygen stimulations.

Furthermore, the kinetic hypoxic response of the intracellular oxygen tension to the extracellular one was investigated. The kinetic response of the cell to the ambient oxygen tension refers to the transient intracellular oxygen tension variation when the oxygen tension varies from low to high (oxygenation) or from high to low (deoxygenation). This kinetics has seldom been studied due to the limited techniques in both microenvironment control and sensing aspects. The variation in the fluorescence intensities within the cells located in regions 1 to 4 during a period of deoxygenation (N_2_ OFF to ON) and reoxygenation (N_2_ OFF to ON) processes is plotted in Figure 5e. The plots show that the larger difference between the fluorescence intensity deviation between deoxygenation and reoxygenation occurs when the extracellular oxygen tension cycles between a higher hypoxia level and normoxia. For example, the deviation in fluorescence intensities of the intracellular oxygen tension-sensitive dye within the AoSMCs and A549 cells located in region 1 was larger than those located in regions 2, 3, and 4. The results suggest that the cells took a longer time to respond to the extracellular oxygen tension variation with a larger range. The transient and kinetic hypoxic response of the cells to the extracellular oxygen variation has seldom been systematically studied due to the technical limitations. The demonstrated results shown in this paper confirm the functionality of the developed device, and it can be further applied to investigate underlying mechanisms when the cells face various oxygen microenvironments.

### 3.3. Intracellular Calcium Level Characterization

For the observation of intracellular calcium levels under cyclic oxygen gradients, both AoSMCs and A549 cells were cultured under cycles of 10 min normoxia (N_2_ OFF)/10 min oxygen gradient (N_2_ ON) for 60 min. During the oxygen tension change, the calcium level was monitored via imaging of the fluorescent active intracellular calcium indicator X-Rhod-1. In the Supplementary Information, Figure S2a,b show the phase contrast and fluorescence images of the AoSMCs and A549 cells stained with the indicator after the experiments, respectively. The images show that no apparent cell morphology change was observed for both cell types after the measurements.

**Figure 6:**
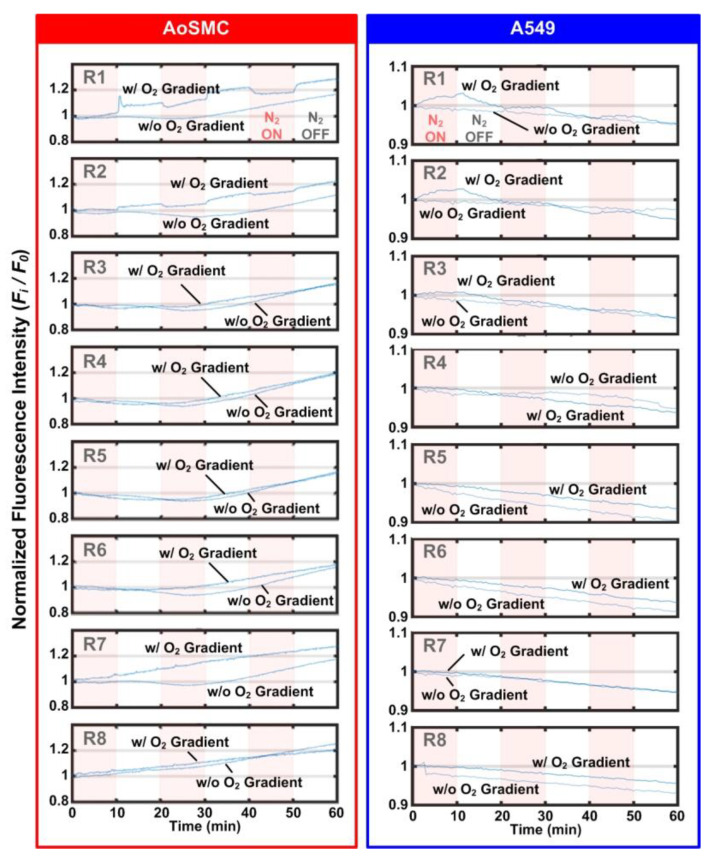
Temporal variation in intracellular calcium within the AoSMCs and A549 cells located in different regions (R1 to R8) based on the fluorescence intensity observation under normoxia (N_2_ OFF) and oxygen gradient conditions (N_2_ ON). Red background regions indicate the periods when nitrogen gas is on (N_2_ ON) for the oxygen gradient generation.

Figure 6 shows the quantitative analysis results of the intracellular calcium concentration variation in the AoSMCs and A549 cells located in different regions under the normoxia and cyclic oxygen gradient stimulation. For AoSMC, the cells cultured under both conditions showed an increasing cytosolic calcium concentration during the 60 min culture inside the microfluidic devices. In addition, the cells located at R1 and R2 exhibited detectable intracellular calcium fluctuation under cyclic oxygen gradient stimulation. When the cellular environment was reoxygenated, i.e., when introducing the mixed air into the left side channel (N_2_ ON to OFF), an increase in the intracellular cytosolic calcium concentration was observed. In contrast, the intracellular cytosolic calcium concentration decreased when the cellular environment was deoxygenated, i.e., when the pure nitrogen was introduced into the side channel (N_2_ OFF to ON).

In comparison, the A549 cells cultured under both conditions showed a slightly decreasing cytosolic calcium concentration during the 60 min culture inside the microfluidic devices. Although the A549 cells located in the R1 and R2 also showed detectable intracellular calcium concentration fluctuation, the amplitude of the variation was smaller than that observed for the AoSMCs. Interestingly, when the cellular environment was reoxygenated and deoxygenated, a decrease and increase in the intracellular cytosolic calcium concentration were observed, respectively. The trends are opposite to those observed for the AoSMCs, and the discrepancy has never been discussed in the literature before and requires further study.

Even though it is not conclusive whether this calcium fluctuation observed on the AoSMCs contributes to muscle movements directly or not, it sheds some light on the possibility that oxygen tension-dependent or feedback regulatory mechanisms exist on the AoSMCs, which has been considered solely an actuator orchestrated by endothelial cells and paracrine signaling [23]. For the lung epithelial (A549) cells, oxygen dependent intracellular calcium fluctuation was observed as well, even though the physiological meaning of the phenomenon remains unclear. From the observation of both cell types, it shows that the hypoxia level with extracellular oxygen tension as low as 6% (according to the calibration value for R2) can trigger the intracellular calcium fluctuation for both cell types.

## 4. Conclusions

In this paper, an integrated approach combining microfluidic devices and real-time fluorescence imaging was developed to study cellular hypoxic response under cyclic oxygen gradients. To demonstrate the capability of the approach, we measured the intracellular oxygen tension and calcium levels of two distinct human cells (AoSMC and A549 cell) and discussed their differences. The intracellular oxygen tension measurement indicated that, even though extracellular and intracellular oxygen tension is closely related, different types of cells can still exhibit different intracellular oxygen tension values in certain extracellular oxygen tensions (4% to 8%) that is not necessary to be the normoxic and hypoxic values (20.9 and 1%, respectively) conventionally recognized. The intracellular calcium tension measurement for AoSMCs show that the reoxygenation from hypoxic (<6%) to normoxic (−21%) environments triggers an increase in intracellular calcium on AoSMCs, which implies that AoSMC, instead of being solely a vessel contraction/dilation actuator, has its own standalone roles in blood pressure regulation. With the two demonstrated applications on monitoring intracellular oxygen tension and the calcium level variation, it is confirmed that the real-time imaging can be exploited to study kinetic cellular hypoxic response due to the simple design and great optical transparency of the device. With the demonstrated functionalities and capabilities, the developed method can pave a way to study the seldom explored kinetic cellular hypoxic response which is essential in various physiological and pathological conditions.

## Data Availability

The data that support the findings of this study are available from the corresponding author upon reasonable request.

## Supplementary Materials

The following supporting information can be downloaded at: https://www.mdpi.com/article/10.3390/bios12111031/s1, Video S1: Cyclic Oxygen Gradient Profiles, Figure S1: Photo of the experimental setup to generate cyclic oxygen gradients in a microfluidic device and perform real-time fluorescence imaging, Figure S2: Phase contrast and fluorescence images of (a) AoSMC and (b) A549 cells cultured in the microfluidic devices and stained with X-Rhod-1 dye for intracellular calcium imaging.
