# Supplementary material for: Study Hypoxic Response under Cyclic Oxygen Gradients Generated in Microfluidic Devices Using Real-Time Fluorescence Imaging
Source: Biosensors (Basel). 2022 Nov 17;12(11):1031. doi: 10.3390/bios12111031 (PMC9688408; doi:10.3390/bios12111031)
Supplement: Supplementary file 1 [file biosensors-12-01031-s001.zip › biosensors-2024385-supplementary.pdf]

Supplementary Information

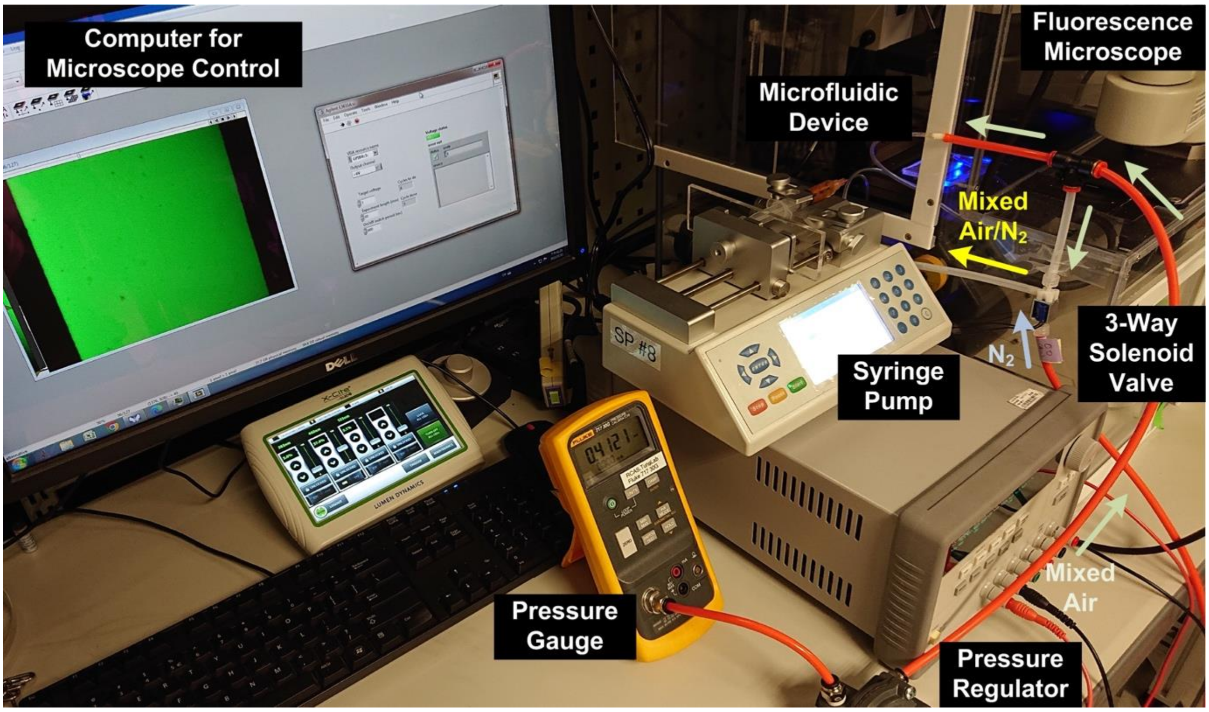

Figure S1. Photo of the experimental setup to generate cyclic oxygen gradients in a microfluidic device and perform real-time fluorescence imaging.

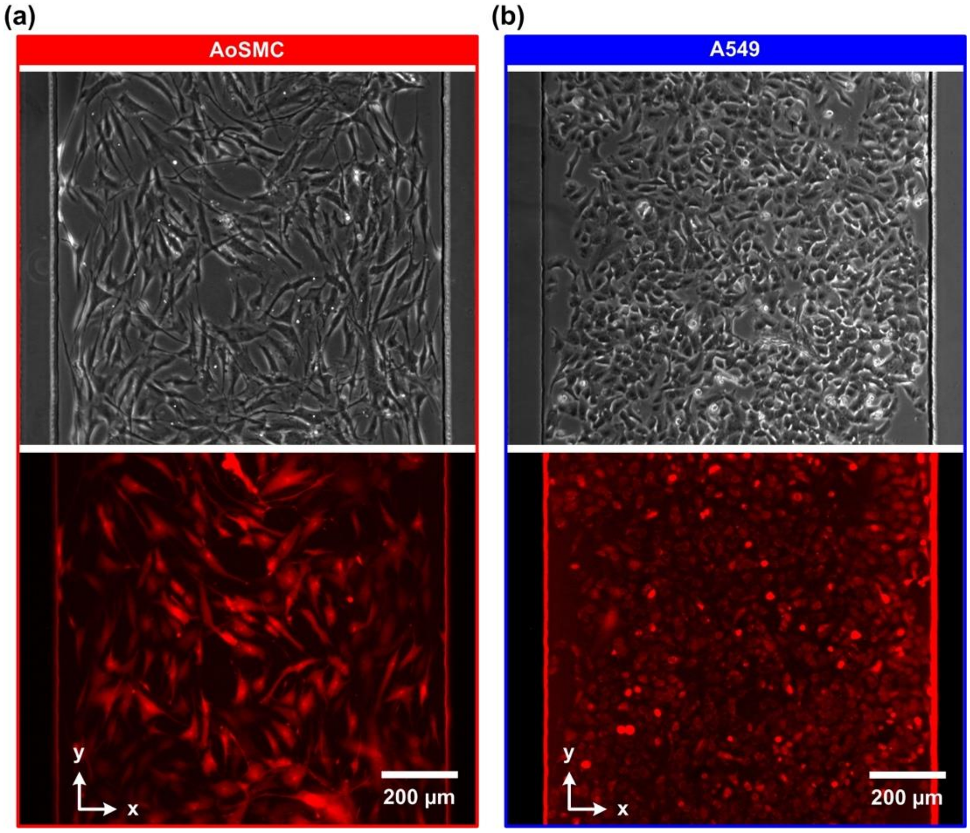

Figure S2. Phase contrast and fluorescence images of (a) AoSMC and (b) A549 cells cultured in the microfluidic devices and stained with X-Rhod-1 dye for intracellular calcium imaging.
